# OP65 The RedETSA Health Technology Assessment Reports Adaptation Tool

**DOI:** 10.1017/S0266462325101220

**Published:** 2025-12-29

**Authors:** Ana Pérez Galán, Alicia Alemán, Perla Mordujovich, Cristian Matías Dorati, Malen Hollmann

## Abstract

**Introduction:**

Adapting health technology assessment (HTA) reports developed by other institutions is efficient to avoid duplication of effort, foster collaboration and knowledge exchange internationally, and improve efficiency. Similar challenges can be faced by institutions who can benefit from experience sharing. A tool for adapting HTA reports in the Americas region was proposed by the Health Technology Assessment Network of the Americas (RedETSA).

**Methods:**

The RedETSA adaptation tool was developed by the RedETSA Working Group on Adaptation of HTA Reports with the participation of 14 people from different countries in the region. To develop this tool, the EUnetHTA tool—translated to Spanish and adapted by the Working Group of the Rapid Reporting Guide for HTA of the Spanish Network of Agencies for HTA and Services of the National Health System (RedETS)—was used as a basis. An iterative process of review, design, and decision was conducted by the entire RedETSA Working Group to develop and finalize the RedETSA tool.

**Results:**

The proposed procedure for adapting reports includes two stages: (i) identification of HTA reports to adapt using different search engines; and (ii) evaluation of the HTA report for adaptation. The proposed tool for evaluating the report to adapt includes a set of questions organized around three modules: module one is a rapid screening on initial operational aspects of the report; module two questions the quality of the report, including the use of technology in the context of the report, efficacy, effectiveness, and safety domains; and module three reviews the feasibility of its application to the local context.

**Conclusions:**

Adapting HTA reports promotes inter-country cooperation, strengthens technical capacities, and avoids duplication of effort. A variety of aspects determines whether a report is suitable or not for adaptation. This proposed tool should be considered as a support guide, subject to local criteria in decision-making, and not a quantitative tool with prescriptive cut-off points.

